# “You Are Not Alone”–Opportunities and Challenges for University Students’ Collaborative Engagement When Dealing With Online Information About COVID-19

**DOI:** 10.3389/fpsyg.2021.728408

**Published:** 2021-10-05

**Authors:** Elisabeth Mayweg-Paus, Maria Zimmermann, Claudia Lefke

**Affiliations:** ^1^Digital Knowledge Management in Higher Education, Institute for Educational Studies, Department of Educational Studies, Humboldt-University of Berlin, Berlin, Germany; ^2^University of Duisburg-Essen, Duisburg-Essen, Germany

**Keywords:** online engagement with scientific information, collaborative learning, information about COVID-19, cognitive and emotional engagement with online information, online support seeking, information seeking abilities

## Abstract

In view of the COVID-19 pandemic, students had to cope with the challenging situation of handling a vast amount of potentially conflicting online information while staying informed. Reading conflicting scientific information has been shown to require cognitive effort for one to integrate it successfully, but reading such information during a crisis–such as the COVID-19 pandemic–may cause additional emotional stress, as students also had to cope with critical aspects of the pandemic (e.g., physical distancing and uncertainty). Different studies have indicated that in crises, stress can be relieved by seeking online social support (as a coping strategy). Similarly, working together (as collaborative learning) can also help people more critically discuss information on a cognitive level. Based on the approaches of online collaborative learning and online social support seeking, we were interested in whether an individual vs. collaborative communication setting would lead to any differences in students’ cognitive as well as emotional engagement with conflicting information about COVID-19. In a 2 × 2 mixed design, *N* = 109 education science students were exposed to two conflicting texts regarding COVID-19 testing that contained current scientific information. The online experiment was conducted in Germany in April 2020, which was the beginning of lockdown in that country. After reading the two texts, participants were asked to reflect on their engagement with the conflicting information either individually (individual group, *n* = 49) or via chat collaboratively (collaboration group, *n* = 60 in 30 dyads). With respect to participants’ written reflections (content-analyzed regarding cognitive as well as emotional engagement), participants in the collaborative group, compared to those in the individual group, more often discussed the pandemic in general and less often engaged emotionally when discussing the evidence from texts. All participants reported higher perceived information overload, lower self-efficacy in sourcing information about COVID-19, and higher active coping strategies after the reflection task compared to before reading the information, with no significant differences between the collaborative and individual groups. We discuss these findings regarding any opportunities and challenges that arise in online collaboration between students for cognitive and emotional engagement when handling conflicting information about COVID-19.

## Introduction

In spring 2020, the COVID-19 pandemic affected various life contexts including educational institutions like universities, which were immediately forced to react to the challenges by shifting their activities to the digital sphere (Adedoyin and Soykan, 2020; Watermeyer et al., 2020). This had major consequences for students (Day et al., 2021): First, students could not go about their normal social lives at university, and instead they had to deal with social isolation, which can evoke stress-related emotions and reduce well-being (Beaunoyer et al., 2020; Miller, 2020; Osimo et al., 2021). Furthermore, regarding their degree of digital readiness, learning in a purely digital environment can be challenging for students and affect their emotional perceptions, resulting in overload, worries, and social and emotional loneliness (Händel et al., 2020).

Additionally, under these pressing circumstances, students have been confronted with a vast amount of science-related online information regarding COVID-19 that can lead to confusion, stress, or disinformation (Ferrara et al., 2020), especially because during the beginning of the pandemic, information concerning the virus was rather vague and diverging, since no one had expertise or experience in dealing with this new situation (Nagler et al., 2020). Furthermore, as shown in a study by Mason et al. (2017) dealing with conflicting science-related information has the potential to elicit physiological stress reactions in students. Apparently, integrating science-related information–such as on COVID-19–seems to be characterized by cognitive efforts and, at the same time, might also include affective reactions. However, while considerable research is concerned with the constraints and affordances of cognitively engaging with scientific information (e.g., List and Alexander, 2017; Hendriks et al., 2020), the emotional processing and emotional effects that online information might have on students require further investigation.

In this context, dealing with science-related online information should not only be viewed as an individual challenge but can also be approached in communication with others, such as in social media contexts. Indeed, studies have shown that students sought help and support from others in online contexts to cope with stress and negative emotions during the pandemic (Eden et al., 2020). Moreover, previous research has highlighted the role of collaborative interaction for cognitive elaboration and critical reflection of science-related online information (Zimmermann and Mayweg-Paus, 2021). However, in the specific situation of university students dealing with online COVID-19 information during physical isolation, collaborative exchange might also serve emotional regulation.

The present study aims at gaining a better understanding of university students’ cognitive and emotional engagement with conflicting COVID-19 information. As the European Digital Competence Framework suggests, being able to evaluate and deal with online information is a crucial component of media competence (Carretero et al., 2017) that needs to be addressed in all areas of formal education (such as in schools or higher education). Further, the specific conditions at the beginning of the lockdown in April 2020 in Germany allow us to analyze students’ behaviors and skills not only on the cognitive level but also on the emotional level, as we can investigate the role of (socio-)emotional dimensions in the processing of science-related online information. Drawing on the literature on how people deal with online scientific information, collaborative argumentation, and (socio)-emotional coping, we strive to examine how university students handle COVID-19 information on the cognitive as well as on the emotional level. This study compares individual vs. collaborative reflection to identify the opportunities and challenges surrounding students’ cognitive as well as emotional engagement with conflicting online information about COVID-19.

### Dealing With Online Scientific Information About COVID-19

In the following, we will first provide literature that describes persons’ individual engagement with online information and, in particular, how dealing with any experienced information overload and conflicting information about COVID-19 might require both cognitive and emotional effort.

When sourcing scientific online information (i.e., seeking, evaluating, and using online information about science-related topics: e.g., Zimmermann and Mayweg-Paus, 2021), students need to handle a vast amount of complex and uncertain information. Online, many possibly relevant information sources exist that also vary in format (e.g., text or video), in genre (e.g., scientific or journalistic), in their explanatory power (e.g., relevance or scientificness), or in their interconnectivity to other online documents (e.g., when a medical expert is interviewed by conspiracy-affiliated news sites) (Goldman and Scardamalia, 2013).

During the COVID-19 pandemic, many people experienced *information overload* (Hong and Kim, 2020; Mohammed et al., 2021), which in health information-related contexts is often defined as the feeling of being overwhelmed by the sheer amount of information (Jensen et al., 2014). Importantly, information overload can also be felt when information about COVID-19 is provided offline via broadcast–in this case, people have little control over what information they take in compared to when they seek information on social media (Mohammed et al., 2021). During the pandemic, people often consumed information from several sources–such as broadcast in addition to social media–and often on a daily basis or even every minute, which can increase feelings of information overload (Hong and Kim, 2020; Motta Zanin et al., 2020; Mohammed et al., 2021). One’s perceived information overload can also be specifically related to one’s actions: For instance, students and university staff who felt overwhelmed by the amount of information they read on COVID-19 also felt less self-efficient in terms of taking measures to avoid COVID-19 (Farooq et al., 2020).

However, these challenges faced by people during the pandemic do not merely encompass the amount of information nor the frequency of retrieving information. Additionally, misinformation was spread on social media, such as that ingesting bleach might help kill the virus (Gharpure et al., 2020). In an analysis of 69 videos about COVID-19 on YouTube, Li et al. (2020) identified that more than 25% of the videos contained misleading information. Additionally, during the pandemic, information seekers were often confronted with the fact that scientific findings may be provisional and open to scientific discussion, such as when scientists openly disagreed with statements by the WHO about the effectiveness of wearing face masks to protect against COVID-19 (Howard et al., 2020). Accordingly, processing (conflicting) online scientific information requires cognitive effort in order to derive appropriate decisions for one’s personal life, such as whether to wear a mask in public.

This is in line with the assumptions of the MD-Trace (multiple-document task-based relevance assessment and content extraction) model (Rouet and Britt, 2012), which describes individuals’ cognitive efforts when processing multiple pieces of information such as in online contexts. The model describes how cognitively demanding it might be to successfully integrate multiple pieces of information; for instance, someone would need to mentally represent the read information together with their meta-information (information about the source, rank of the search result, or interconnectedness to other information, especially if information conflicts) in order to evaluate the quality of information appropriately. In this sense, evaluating a single piece of online information is a complex process that requires people not only to assess the information based on whether it is complete, correct, and appropriate but also to identify whether they can rely on the provider of the information (Bromme and Goldman, 2014; Bråten et al., 2014).

However, when engaging with online information, other processes are at play in addition to cognitive ones. Regarding research approaches to examine multiple text comprehension (e.g., MD-Trace model: Rouet and Britt, 2012), the cognitive affective engagement model of multiple source use (CAEM) (List and Alexander, 2017) describes students’ cognitive as well as emotional engagement when reading different texts. It, thus, expands approaches that focus on the cognitive handling of multiple information with important motivational aspects (such as one’s personal relevance to seek appropriately). In the context of COVID-19, interest in the topic and other motivational aspects become evident as COVID-19 poses various risks to one’s personal health: In addition to the cognitive effortful evaluation of the mere complexity of online scientific information, a student assessing COVID-related information would need to evaluate what personal or societal risks any decision may entail. According to research that considers cognitively more effortful vs. more effortless processes of dealing with information (e.g., the information seeking and processing model: Griffin et al., 1999; or trust in online information: Metzger and Flanagin, 2013), during the high-risk situation of the pandemic, students’ uncertainty likely drove them to use more cognitive effort when engaging with information. With respect to citizens’ uncertainty during the pandemic, initial studies reported that during March 2020 (at the beginning of the pandemic) in Italy, more than 30% of surveyed citizens reported feelings of uncertainty (Motta Zanin et al., 2020). However, while the perception of high risk may lead people to cognitively effortfully engage with (conflicting) scientific information, such risk perception and conflicting information may also cause confusion, anxiety, and stress (e.g., Mason et al., 2017; Li and Lyu, 2021; Oyetunji et al., 2021). Further, strong emotional reactions (e.g., stress) occurring while reading conflicting information about COVID-19 may have affected students’ sourcing abilities (e.g., their ability to say who had said what, which is crucial to coherently represent conflicting information: Bråten et al., 2014) (Mason et al., 2017).

Importantly, the described challenges one might face while cognitively and emotionally engaging with online information may become particularly relevant during the pandemic, since exposure to COVID-19 misinformation may lead people to avoid information (Kim et al., 2020) or may even cause health problems. Hence, it is particularly important that students can engage with online scientific information about COVID-19 competently. As such, students need to overcome certain challenges when sourcing online information about COVID-19. Further, engaging with such information may require effort, so to be successful students may depend on their *self-efficacy in sourcing online information* (Andreassen and Bråten, 2013; Caena and Redecker, 2019; Hendriks et al., 2020; Zimmermann and Mayweg-Paus, 2021). According to Bandura (1997), self-efficacy refers to one’s belief in one’s own capabilities to organize and execute the courses of action required to attain particular goals. Thus, students’ self-efficacy in sourcing online information about COVID-19 reflects how they interpret their own competencies around sourcing such information (Kurbanoglu, 2003). While self-efficacy in sourcing information is considered an important aspect of students’ digital competence (Carretero et al., 2017), initial studies empirically indicate that students’ self-efficacy in sourcing online information is actually related to their skillful sourcing behavior: For instance, students who had higher self-efficacy used online library databases rather than Google to search for information (Tang and Tseng, 2013).

### Collaborative Engagement When Dealing With Online Information

Considering that dealing with online information requires effort and skill, here we discuss how engaging with online information in a collaborative manner (with other people) might help students to reflect on their cognitive as well as emotional engagement with online information.

With regard cognitive levels during learning processes, collaborative interaction used in learning and skill development has been shown to be beneficial for various educational contexts (Chen et al., 2018). In particular, collaborative engagement seems to provide a promising setting for sharing, interpreting and critically examining scientific/science-related information in online contexts (Hendriks et al., 2020). In such contexts, a person is subject to others’ scrutiny of their own position, which, in turn, enhances one’s need to be more critical not only toward one’s own position but also the opposing position. As research shows, the dialogic nature of the interaction directly affects how people handling evidence: In a collaborative setting, students seem to use evidence more often to address opposing viewpoints in an elaborated way, whereas in an individual setting they are more likely to stick to the information given to them (shared evidence) instead of integrating new information (Kuhn and Moore, 2015; Mayweg-Paus and Macagno, 2016). The potential of collaborative engagement for helping people deal with scientific online information efficiently lies in specific communicative moves (such as exchanging multiple perspectives) that can elicit (deeper) cognitive processing of information and a critical reflection of sources. Furthermore, collaborative engagement fosters critical elaboration not only of the information itself but also of the sources from where such information comes: In a study on students’ critical reasoning about their own information sourcing strategies, the students who worked collaboratively reasoned more frequently in an elaborated way about the information and their selection of information than the students who worked individually. At the same time, however, in that study, students’ self-efficacy in sourcing online information increased after both forms of reasoning, namely students’ individual reasoning as well as collaborative reasoning about their own selection (Zimmermann and Mayweg-Paus, 2021). This means that individual and collaborative engagement with online information are both advantageous for activating reflection about one’s sourcing skills.

Thus, studies have indicated that, in contrast to individual reflection settings, collaborative reflection settings may be beneficial in order to promote students’ elaboration of their own thinking and challenging of the other’s idea (Kuhn and Moore, 2015), their elaborated reasoning of their own sourcing strategies (Zimmermann and Mayweg-Paus, 2021), as well as their analysis and reflection of problems (Csanadi et al., 2020). However, in direct comparisons of individual and collaborative communicative settings wherein students engaged in cognitive reasoning processes, dyads have been shown to spend more time and effort than individuals: Collaboration requires time to explain possible causes of a problem (Csanadi et al., 2020); in collaboration there is a risk that some partners may move more quickly through the phases of the collaboration task, before everyone is ready (Mullins et al., 2011); and in collaborative settings, partners spend more time on managing the task (Zimmermann and Mayweg-Paus, 2021).

Besides the role of cognitive processing of science-related online information in collaborative engagement, the particular situation of dealing with COVID-19-related information while interacting with others should also serve functions on socio-emotional levels (i.e., the socio-relational, socio-emotional, and motivational aspects of dialogs) that often have been largely neglected in research on collaborative learning (Asterhan, 2013). In this sense, students may profit from the collaboration in at least two ways: (1) They may deal better with the information itself, allowing them to find the “best” solution–especially in dialogs with collaborative goal orientation that cause speakers to take a cooperative stance on what they see as a shared enterprise (e.g., Asterhan, 2013), and (2) they may support each other in coping with the (potentially highly negative) emotions the information might cause.

During the pandemic, students had to face several negative emotions such as feelings of loneliness, fear, and stress triggered by physical isolation during lockdowns, quarantines, or the measures implemented by governments (Wang et al., 2020; Awoke et al., 2021). A study at universities in the Philippines showed social and emotional support are important factors for reducing these negative feelings (De Los Santos et al., 2021).

Searching online for social and emotional support is often considered to fall under the concept of *online support seeking as coping strategies*. According to Zimmer-Gembeck and Skinner (2016, p. 2) *coping* is “a fundamental human adaptive process that involves the regulation of multiple subsystems (e.g., emotion and attention) that are activated by stress and that also show regular age-graded developments in how such regulation is accomplished.” While research has investigated hundreds of ways people cope (Skinner et al., 2003), they are often categorized into two common types (Zimmer-Gembeck and Skinner, 2016), namely *active strategies* and *avoidance strategies*. Active strategies involve cognitive and behavioral processes that actively respond to the situation that cause stress (e.g., seeking emotional support or positive reappraisal of the situation). In contrast, avoidance strategies are characterized by disengagement or passive responses toward the stressor (e.g., distraction from the problem, cognitive avoidance, and social withdrawal). Often, the avoidance strategies are associated with more negative outcomes, such as distress (Zimmer-Gembeck and Skinner, 2016).

Importantly, due to the special situation during the COVID-19 pandemic, most students were only able to seek support in online contexts. van Ingen et al. (2016, p. 512) defined online coping as “thoughts and behaviors facilitated by the Internet that people use to manage stressful situations.” An example of online coping is seeking social support in social network services (SNS), such as an online support group. Such active forms of coping can empower people in many ways: They may help one get important information (informational support), and they may help people express emotions and share experiences (emotional support) (Barak et al., 2008). Social support, as one of many coping strategies, is characterized by forms of collaborative support in the emotional as well as the cognitive processes one faces during a stressful situation (e.g., when reading conflicting information about COVID-19 during the pandemic). In such a context, one study at universities in Italy has shown that the feeling of togetherness and the feeling of being a part of an academic community, especially in the COVID-19 pandemic, reduced perceived stress (Procentese et al., 2020).

However, current research on coping online in the context of SNS indicates that the mere use of SNS may also induce stress and may lead to emotional exhaustion or even increase the perceived information overload (Lim and Choi, 2017; Hong and Kim, 2020). Nonetheless, users who have high levels of coping resources (such relying on others to make them feel better or trying to get advice) have shown they can better manage the stress brought on by SNS (Lim and Choi, 2017).

To our knowledge, so far no research has investigated whether collaborative engagement with conflicting information has any impact on students’ active and avoidance coping strategies in terms of engaging with such information. However, during the difficult time at the beginning of the pandemic, students may have been able to overcome the challenges associated with online information by collaborating with others as well as seeking social support when reading information about COVID-19 (e.g., regarding how to decide whom to trust and what information to rely on).

### Rationale for the Study

During the beginning of the pandemic, students’ success in sourcing information about COVID-19 was confronted with various challenges, such as their perceived overload of information about COVID-19 (e.g., Hong and Kim, 2020), their emotional reactions caused by any conflicting information about COVID-19 (e.g., Mason et al., 2017; Ferrara et al., 2020), as well as any feelings of loneliness or anxiety at large, which may have been caused, for example, by government measures instituted to prevent the spread of the virus (Beaunoyer et al., 2020; Miller, 2020; Awoke et al., 2021). Accordingly, in this study we investigate students’ engagement with conflicting information about COVID-19 by considering not only indicators for their cognitive engagement but also for their emotional engagement with this information. As research on online collaboration and online social coping indicate that collaboration in this manner offers opportunities as well as challenges for both students’ cognitive and emotional engagement with scientific online information, this study further aims at investigating the opportunities and challenges related to collaborative communication settings wherein students can reflect together with someone else about how they engage with conflicting information on COVID-19 tests.

Based on research approaches to collaboration and argumentation, collaborative argumentation settings tend to encourage people to engage with evidence more reflectively and in a more differentiated way (e.g., Chin and Osborne, 2008; Kuhn and Moore, 2015; Mayweg-Paus and Macagno, 2016), which may help students critically question the evidence presented in the conflicting information (e.g., Mayweg-Paus and Macagno, 2016; Mayweg-Paus et al., 2016). However, individual reflection settings may also have advantages, as they are much more easy to manage and may help students reflect on their sourcing competencies (Mullins et al., 2011; Zimmermann and Mayweg-Paus, 2021). This leads to our first research question:

RQ1: How do students cognitively engage with conflicting information about COVID-19 in an individual compared to a collaborative reflection setting, and how does the setting affect students’ perception of information overload, their self-efficacy in sourcing COVID-19 information, and their communicative reflection behavior?

However, since during the pandemic it was likely that students did not engage with information purely rationally, detached from any emotional reactions, this study also focuses on students’ emotional engagement with the information (Mason et al., 2017) as well as on whether collaboration as applied to seeking social support has an impact on their perceived coping strategies (Lim and Choi, 2017). Given the research on online support seeking as a coping strategy, taking part in a community and receiving forms of social support may prevent feelings of stress and loneliness during a crisis (e.g., the pandemic) (Procentese et al., 2020; De Los Santos et al., 2021); but, online coping may also induce stress or even increase the perceived information overload (Hong and Kim, 2020). This leads to our second research question:

RQ2: How do students emotionally engage with conflicting information about COVID-19 in an individual compared to a collaborative reflection setting, and how does the setting affect students’ perceived coping strategies as well as their communicative reflection behavior?

Hence, to gain deep insights into the potential opportunities and challenges of either collaborative or individual reflection settings, this study focuses on students’ cognitive as well as their emotional engagement with COVID-19 information as well as on various quantitative and qualitative indicators of their success in engaging with information about COVID-19.

## Materials and Methods

### Participants

Overall *N* = 122 students initially participated in the study voluntarily and were reimbursed with 15 euros. Participants were studying various disciplines, at either the bachelor’s or the master’s level. They were recruited from different German universities via email lists. We excluded data from *n* = 13 participants whose Internet connectivity failed during the investigation. Hence, we analyzed data from *N* = 109 participants (73 female and 2 diverse) aged 18 to 48 (*M* = 24.13, *SD* = 5.08), with *n* = 49 participants in the individual group (group_*in*_) and *n* = 60 participants in the discourse group (group_*coll*_) (paired in *n* = 30 dyads). Of these 109 participants, 103 indicated German as their first language. Those six participants whose first language was not German had been speaking German for on average *M* = 11.33 years (*SD* = 7.69). At the time of the investigation, participants had been studying for an average of *M* = 4.12 semesters (*M* = 2.06 years) (*SD* = 2.52). *N* = 39 from the group_*co*__*l*__*l*_ and *n* = 39 from the group_*in*_ were studying at the bachelor’s level, while *n* = 10 from the group_*coll*_ and *n* = 21 from the group_*in*_ were studying at the master’s level. In terms of participants’ gender, *n* = 37 participants in the group_*coll*_ and *n* = 36 in the group_*in*_ were female (differences between experimental conditions were not significant; study level: χ^2^(1) = 2.82, *p* = 0.09; and gender: χ^2^(2) = 3.88, *p* = 0.14). The average duration of participation for all participants was *M* = 48.49 min (*SD* = 17.85), and this duration did significantly differ between the experimental conditions (group_*coll*_: *M* = 55.6, *SD* = 16.37; group_*in*_: *M* = 42.87, *SD* = 17.09), *F*(1, 84) = 3.5 = *p* = 0.001.

Participants reported that they used a computer, notebook, or tablet for an average of *M* = 4.21 (*SD* = 2.78) hours per day and spent on average *M* = 5.66 (*SD* = 3.45) hours per day on the Internet. Participants reportedly sought general online information for an average of *M* = 2.20 (*SD* = 1.51) hours per day and searched specifically for online information about COVID-19 for an average of *M* = 0.84 (*SD* = 2.45) hours per day, with no significant differences between experimental conditions, all *F*(1, 100) ≤ 1.82, *p* ≥ 0.18, η^2^ ≥ 0.0.18).

Regarding their prior knowledge about COVID-19, participants gave a score, on average, of *M* = 3.51 (*SD* = 0.86), meaning that they reported to have rather high knowledge (four items: e.g., “I often do research about the topic COVID-19 on the Internet”). Furthermore, on average all participants reported to find the COVID-19 measures by the government as relevant (*M* = 3.91, *SD* = 0.62) (four items: e.g., “I think the measures are reasonable.”) (all eight items used five-point Likert scales, with 1 = strongly disagree to 5 = strongly agree). Lastly, all participants reported that they sought support on social media a “few times” to “from time to time” (*M* = 2.65; SD = 0.81) (four items: e.g., “How often do you seek support for issues in your preferred social media networks.”) (items were on a five-point Likert scale, with 1 = never to 5 = very often).

### Design

In a 2 × 2 mixed design, with the between-participants factor experimental condition (individual vs. collaborative reflection) and the within-participants factor time (pre- vs. post-measure), we assessed the participants’ self-reported (1) information overload, (2) information sourcing self-efficacy, and (3) active as well as avoidance coping strategies before and after the reflection task. Participants were instructed to individually read two conflicting texts on “tests for COVID-19.” Afterward, all participants were asked to reflect about how they engage with such conflicting information about COVID-19. Participants were randomly assigned to one of the two experimental conditions (i.e., individual vs. collaborative reflection task). After reading the two texts, participants in the groupin individually reflected, while participants in the groupcoll engaged in a collaborative discourse via chat and reflected collaboratively about how they engage with such conflicting information (see Supplementary Material 1 for the instructions). In the groupcoll, they were randomly paired into 30 dyads.

### Procedure

Participants performed the experiment online at any place where they could connect to the Internet on their own digital device. Each participant was invited to take part in the online experiment via email list invitations. Before the experiment, all participants were introduced to the experimenters via video call application (i.e., https://www.edudip.com/de), where they received information about how participants can conduct the online survey and, for the collaborative group, how they can chat with each other. During the experiment participants worked at their own pace and were guided through the experiment by the online survey (unipark.com by Questback EFS Surveys).

First, participants answered items relating to demographic variables. They were then asked to report their perceived information overload about COVID-19 information, their self-perceived COVID-19 information sourcing self-efficacy, as well as their coping strategies when reading unpleasant information (pre-measure). Afterward, participants received a fictional scenario: They were asked to imagine themselves searching for information on the topic of “tests for COVID-19” and finally finding two online articles. All participants were instructed to read the same articles. The groupcoll was further instructed to subsequently discuss these search results with another person. The participants in the groupcoll had to open a window of the open-source chat application (i.e., https://discordapp.com/) for chatting with another participant. The participants were asked to reflect–either individually or collaboratively–on how they engage with such information about tests for COVID-19. Afterward, all participants were again asked to rate their perceived information overload, their self-perceived information sourcing self-efficacy, and their coping strategies (post-measure).

### Materials

We created the two online articles regarding tests for COVID-19 in order to control for any aspects that may have influenced how students judged the credibility of the texts (e.g., expertise of the provider of information, technical terminology, or one-sided vs. two-sided argumentation of providers; e.g., Mayweg and Jucks, 2017; Zimmermann and Jucks, 2018). As such, we included information that came from real online articles published at the end of March 2020 in connection with COVID-19 testing. Both texts entailed the same amount of scientific information and referred to actual scientific evidence about COVID-19 tests with information about what was known at the time the experiment was conducted. The information summarized in the two articles reflected the findings of two scientific studies about COVID-19 tests. Both texts were provided by supposed medical doctors. Both providers explained that the tests were either valid or not valid and drew different conclusions about the social measures during the pandemic derived from the tests (see Supplementary Materials 2, 3).

### Measurements

#### Communicative Reflection Behavior

To assess participants’ communicative reflection behavior as an indicator for participants’ cognitive and emotional engagement with conflicting information about COVID-19, we analyzed their communicative behavior in the reflection task (i.e., individual and collaborative reflection about their engagement with the conflicting information about COVID-19 tests). Participants’ reflection behavior was divided into units of meanings, where each unit contained a participant’s semantic description of a distinct theme or idea (Clarà and Mauri, 2010). To code the units of meanings (coding scheme described below), a rater assessed all of the *N* = 78 texts that emerged from the reflection task. The level of agreement between two independent raters, in terms of *n* = 21 (26.6%) of all texts (randomly and equally distributed from the *N* = 49 individual and *N* = 30 collaborative texts), was Cohen’s Kappa = 0.94. The percentage of agreement between these two independent raters was PA = 87.32–100% for all coding at the level of the categories. Furthermore, the coders’ coding reached 100% agreement for 12 out of the 21 documents at the levels of the units of meanings.

From participants’ individual and collaborative communicative reflection behavior, we determined the relative frequencies of the coding categories that were discussed by participants in relation to the overall frequencies of on-task units (i.e., comments made about the reflection task). These relative frequencies thus represent the relative numbers regarding all task-related comments and not the total number of comments made (there were also off-task comments, e.g., related to the management of the reflection task).

The coding scheme aimed to describe how participants reflected (1) cognitively and (2) emotionally about their engagement with the conflicting information and about evidence and information from and beyond the read information. In terms of participants’ cognitive engagement, the first coding category relates to participants’ discussion about evidence read in the presented information, (i.e., regarding the credibility of information, such as whether the information was considered scientific or recent, and the trustworthiness of the providers, such as whether they are benevolent and competent: Bromme and Goldman, 2014). Furthermore, also related to participants’ cognitive engagement, the second coding category relates to comments about how they deal with evidence beyond the information they read (i.e., when participants mentioned or questioned other aspects relevant for their engagement with evidence, such as the scientificness of evidence, the authority behind the evidence, anecdotal-related evidence, or how they deal with the credibility of information in general: Bromme and Goldman, 2014; Mayweg-Paus and Macagno, 2016). Furthermore, participants also discussed the pandemic in general without providing any evidence at all (e.g., they discussed pandemic-related measures: “I think consequences should have been implemented sooner”). Hence, in a third coding category, we coded participants’ comments related to discussions about the pandemic in general.

In terms of participants’ emotional engagement, the fourth coding category relates to criteria associated with participants’ discussion on how reading the presented conflicting information affected them emotionally (e.g., when they were confused by the texts) (Mason et al., 2017). Furthermore, with respect to participants’ emotional engagement, we also assessed whether participants emotionally discussed the pandemic beyond the read information (e.g., when they reported anxiety that loved ones might become infected) (e.g., Awoke et al., 2021). Finally, remaining categories referred to comments that did not match the previously reported categories.

The coding scheme derived from participants’ reflection task is given in Supplementary Material 4. Examples of reflective communication behavior for the individual as well as collaborative reflection task are given in Tables 1, 2.

**TABLE 1:** Example of an individual reasoning behavior (translation in brackets).

Code	Person	Comment
Cognitive comment on text	A	- Widersprüchliche Informationen regen an noch mehr Informationen einzuholen. - (Contradicting information encourages you to obtain even more information.)
Emotional comment on text		- Beide Texte klangen an und für sich logisch und in sich schlüssig, jedoch ist es aufwühlend und regt an noch mehr Informationen einzuholen. - (Both texts sounded logical and coherent in themselves, but it is overwhelming and encourages you to get even more information.)
Cognitive comment beyond text		- Ich persönlich würde lediglich aus Sympathieempfinden mich für die weitere Recherche für den 1. Text entscheiden und unterstützende Literaturen suchen. - (Personally, I would decide to research the first text simply out of sympathy and look for supporting literature.)
Emotional comment on text		- Dennoch ist es ein zwiegespaltenes Gefühl/hin und her gerissen wem man bezüglich des COVID-19 trauen kann. - (Nevertheless, it is with mixed feeling/torn back and forth who can be trusted with regard to COVID-19.)

**TABLE 2:** Example of a collaborative reasoning behavior (translation in brackets).

Code	Speaker	Comment
Cognitive comment on text	A	- So, ich bin etwas hin- und hergerissen, was die beiden Artikel angeht. Beim ersten Artikel dachte ich noch: Interessant! Eine Kontaktsperre scheint also sinnvoll, um das Virus wirklich einzudämmen. Der Artikel rechtfertigt ja auch die Verlängerung einer Kontaktsperre. - (So, I’m a bit torn about the two articles. With the first article, I still thought: Interesting! A contact ban seems to make sense in order to really contain the virus. The article also justifies the extension of a contact ban.)
Emotional comment on text	A	- Nach dem Lesen des zweiten Artikels war ich wieder verunsichert und dachte: Okay, die radikalen Maßnahmen sind vielleicht doch nicht so sinnvoll. - (After reading the second article, I was unsure again and thought: Okay, maybe the radical measures are not so sensible after all.)
Cognitive comment on text	A	- Jedoch sehe ich keinen Gegenvorschlag in diesem Artikel, außer weiterzuleben wie gewohnt. Kann das wirklich der richtige Ansatz sein? - (However, I don’t see any counter suggestion in this article, except to continue living as usual. Can this really be the right approach?)
Cognitive comment about the pandemic in general	A	- Ich bin wirtschaftlich nicht besonders bewandert, so dass mir die weitreichenden Konsequenzen der Kontakt- und Berufssperre nicht wirklich bewusst sind. Ich kann persönlich also nicht abwägen, was schlimmer wäre: Wirtschaftliche Folgen oder gesundheitliche Folgen. - (I’m not very economically experienced, so I’m not really aware of the far-reaching consequences of being barred from contact and work. I personally can’t weigh what would be worse: economic consequences or health consequences.)
Cognitive comment on text	B	- Der erste Artikel hat auch eher das wiedergegeben, was man ja schon so kannte: viele asymptotisch (hoffentlich korrekt geschrieben) und somit eine hohe Dunkelziffer. Was ich mir auch gut vorstellen kann. - (The first article also rather reflected what was already known: many asymptotically (hopefully spelled correctly) and thus a high number of unreported cases. What I can also well imagine.)
Cognitive comment on text	B	- Der zweite Text hat mich dann erst darauf aufmerksam gemacht, dass der Test wohl recht unzuverlässig ist. Da würde ich jetzt eher nochmal hinterherrecherchieren, ob das auch tatsächlich so stimmt. - (The second text only made me aware that the test is probably quite unreliable. Now I would rather do more research to find out if this is really true.)
Cognitive comment about the pandemic in general	B	- Wirtschaftliche gegen gesundheitliche Folgen abwägen, ist halt auch schwierig. - (Weighing economic against health consequences is also difficult.)

#### Perceived COVID-19 Information Overload

To assess participants’ perceived information overload when sourcing information about COVID-19 as an indicator for their cognitive engagement with this information, we adapted items to assess health-related information overload by Ramondt and Ramírez (2018) (e.g., “The point has come where I no longer even bother to get the latest information about the corona virus” or “There are so many recommendations about the corona virus, it is difficult to decide which recommendation to follow.”). The items refer to participants’ perceived information overload not only in the context of perceived overload while seeking information but also in the context of perceived information overload when it comes to the evaluation of information about the corona virus in general. The internal consistency for the eight items at the pre-measure was Cronbach’s α = 0.56. At the post-measure, it was Cronbach’s α = 0.53. The removal of any individual item would not have resulted in a significant increase of the internal consistency. The five-point Likert scale ranged from 1 = strongly disagree to 5 = strongly agree.

#### COVID-19 Information Sourcing Self-Efficacy

To assess participants’ self-efficacy when sourcing information about COVID-19 as another indicator for their cognitive and emotional engagement with this information, we adapted items from the Information Seeking Self-Efficacy Scale (IRSES) (Bronstein, 2014: adapted by Hinson et al., 2003). All items focused on the sourcing of COVID-19 information. The scale incorporates three dimensions related to one’s personal self-evaluation [e.g., “If I don’t know how to assess information about the corona virus, I give up quickly.” or “I do not understand most of the information about the corona virus.” (11 items)]; one’s comparison with others [e.g., “Most other people know better than me how to evaluate certain information about the corona virus” (4 items)]; and one’s physical state while seeking [e.g., “I feel stressed when seeking information about the corona virus.” (5 items)]. The consideration of seeking in this scale, hence, refers to this study’s concept of sourcing online information, as the items assess aspects related to seeking information and aspects related to the evaluation and usage of information. The internal consistency for the 20 items at the pre-measure was Cronbach’s α = 0.77. At the post-measure, it was Cronbach’s α = 0.85. The five-point Likert scale ranged from 1 = strongly disagree to 5 = strongly agree.

#### Coping With Information About COVID-19

To assess the participants’ coping strategies when reading information about COVID-19 as another indicator for their emotional engagement, we adapted two subscales from Lim and Choi (2017). The items address participants’ active coping strategies (e.g., “I ask friends for help” or “I ask friends who have similar experiences how they deal with it.”) (seven items) and participants’ avoidance coping strategies (“I avoid thinking about it”) (three items). Participants were asked to state on all items how they handle unpleasant information about COVID-19. Internal consistency at the pre-measure was Cronbach’s α = 0.79. At the post-measure, it was Cronbach’s α = 0.81. The five-point Likert scale ranged from 1 = strongly disagree to 5 = strongly agree.

#### Affective State as Preparatory Variable

Because we carried out the study at a time when the pandemic itself may have strongly influenced the participants’ emotions, it is important to control for any variance among the individual or collaborative reflection that could been caused by the basic current affective state of the participants. Thus, to assess participants’ affective state before reading COVID-19 information, participants reported their affective state based on PANAS (Janke and Glöckner-Rist, 2014). The PANAS is a widely used measurement to assess persons’ affective state, and it uses 20 items (e.g., “interested,” or “attentive”). The PANAS includes two subscales (PANAS-Positive affect and PANAS-Negative affect). The five-point Likert scale ranged from 1 = slightly or not at all to 5 = very much. Internal consistency was Cronbach’s α = 0.88. Participants’ values in terms of the subscale PANAS-Negative affect were on average rather low (i.e., at the beginning of the study, participants were very slightly or not at all to a little, e.g., “scared” or “upset”). On average, participants’ values in terms of the subscale PANAS-Positive affect were *M* = 2.63 (i.e., participants were to some extent, e.g., “interested” or “attentive”) (see Table 3). In a ANOVA with affective state as the dependent variable and the experimental conditions as the independent variable, there were no significant differences between the experimental groups with regard to participants’ affective state, both subscales *F*(1,76) ≤ 0.201, *p* ≤ 0.655, η^2^ ≥ 0.002 (see Table 4). Hence, this variable was not included in our main analysis as a control variable.

**TABLE 3:** Descriptive values in terms of the scales.

Dependent Measure	Experimental Condition	*M*	*SD*
Scale overload pre	Individual	2.56	0.65
	Collaborative	2.49	0.61
	Total	2.52	0.62
Scale overload post	Individual	2.66	0.59
	Collaborative	2.63	0.61
	Total	2.64	0.60
Scale sourcing self-efficacy pre	Individual	3.43	0.51
	Collaborative	3.53	0.54
	Total	3.48	0.52
Scale sourcing self-efficacy post	Individual	3.35	0.54
	Collaborative	3.37	0.59
	Total	3.36	0.57
Scale coping avoidance pre	Individual	3.20	0.78
	Collaborative	3.01	0.75
	Total	3.10	0.77
Scale coping avoidance post	Individual	3.11	0.82
	Collaborative	3.08	0.70
	Total	3.10	0.75
Scale coping active pre	Individual	3.22	0.75
	Collaborative	3.35	0.80
	Total	3.29	0.78
Scale coping active post	Individual	3.27	0.76
	Collaborative	3.59	0.79
	Total	3.45	0.79
Scale PANAS negative affect pre	Individual	1.74	0.70
	Collaborative	1.79	0.71
	Total	1.77	0.70
Scale PANAS positive affect pre	Individual	2.61	0.61
	Collaborative	2.65	0.69
	Total	2.63	0.65

*Individual group *n* = 49; collaborative group *n* = 60; *NTotal* = 109.*

*Relative frequencies in ratio to all on-task units.*

**TABLE 4:** MANOVA to test for differences between the experimental conditions and within subjects (time) regarding the scales.

Source	Measure	Type III Sum of Squares	*df*	*Mean Square*	*F*	*p*	η^2^*part*
Time	Information overload	0.700	1	0.70	8.52	0.00	0.08
	Sourcing self-efficacy	0.685	1	0.69	11.19	0.00	0.10
	Coping avoidance	0.004	1	0.00	0.03	0.87	0.00
	Coping active	1.108	1	1.11	6.34	0.01	0.06
Condition	Information overload	0.100	1	0.10	0.15	0.70	0.00
	Sourcing self-efficacy	0.205	1	0.21	0.38	0.54	0.00
	Coping avoidance	0.642	1	0.64	0.63	0.43	0.01
	Coping active	2.811	1	2.81	2.73	0.10	0.03
	PANAS negative affect (preparatory analysis)	0.187	1	0.19	0.20	0.66	0.00
	PANAS negative affect (preparatory analysis)	0.006	1	0.01	0.01	0.93	0.00
Time × condition	Information overload	0.045	1	0.05	0.54	0.46	0.01
	Sourcing self-efficacy	0.080	1	0.08	1.30	0.26	0.01
	Coping avoidance	0.341	1	0.34	2.56	0.11	0.02
	Coping active	0.454	1	0.45	2.60	0.11	0.02

#### Main Analyses

Two generalized linear models were conducted to test whether participants’ pre- and post-measures for participants’ information overload, information sourcing self-efficacy, active as well as avoidance coping strategies, as well as their relative frequencies of emotional and cognitive engagement in terms of participants’ reflection behavior differed between the experimental conditions. We set an α error of α = 0.01.

## Results

### Results of Participants’ Communicative Reflection Behavior

We conducted a linear model, including the between factor *experimental condition* and the dependent variables *relative frequencies of types of reflection* (i.e., comments about evidence on and beyond text, as well as about the pandemic in general; and emotional comments on texts and in terms of the pandemic in general). Participants in the group_*coll*_ more often discussed the pandemic in general–without providing or discussing any evidence (*M* = 37.1%, *SD* = 25.54)–and less often engaged emotionally when discussing the evidence related to the read texts (*M* = 3.45%, *SD* = 4.62) compared to the group_*in*_ (discussions about pandemic in general: *M* = 15.5%, *SD* = 18.6; emotional discussion about evidence from text: *M* = 12.8%, *SD* = 19.13), both *F*(1,76) ≥ 6.84, *p* ≤ 0.01, η^2^ ≥ 0.08. Participants in both groups discussed evidence from the texts with the same frequency (group_*coll*_: *M* = 33.7%, *SD* = 26.5; group_*in*_: *M* = 33.4%, *SD* = 27.3), *F*(1,76) = 0.002, *p* = 0.97, η^2^ < 0.01). Similarly, there were no significant differences in terms of participants’ cognitive reflection about evidence beyond the text or their comments related to emotions about the pandemic in general (see Tables 5, 6). Putting together all the comments that are characterized either by cognitive or emotional engagement, participants in the group_*coll*_ more often engaged cognitively (*M* = 88.8%, *SD* = 13.5) and less often engaged emotionally (*M* = 11.2%, *SD* = 13.5), compared to the group_*in*_ (cognitively: *M* = 76.9%, *SD* = 20.78; emotionally: *M* = 23.1%, *SD* = 20.8), both *F*(1,76) = 7.73, *p* = 0.007, η^2^ = 0.09.

**TABLE 5:** Descriptive statistics of the relative frequencies of cognitive and emotional reflection around the engagement with conflicting information and the pandemic in general.

Relative frequency of	Experimental condition	*M*	*SD*
Cognitive comments on text	Individual	0.33	0.27
	Collaborative	0.34	0.27
	Total	0.34	0.27
Cognitive comments beyond text	Individual	0.28	0.26
	Collaborative	0.18	0.14
	Total	0.24	0.22
Cognitive comments on pandemic without evidence	Individual	0.15	0.19
	Collaborative	0.37	0.26
	Total	0.24	0.24
Emotional comments on text	Individual	0.13	0.19
	Collaborative	0.04	0.05
	Total	0.09	0.16
Emotional comments beyond text	Individual	0.02	0.05
	Collaborative	0.04	0.10
	Total	0.03	0.07
All off-task	Individual	0.00	0.00
	Collaborative	0.34	0.12
	Total	0.13	0.18
All on-task	Individual	1.00	0.00
	Collaborative	0.66	0.12
	Total	0.87	0.18
All cognitive comments	Individual	0.77	0.21
	Collaborative	0.89	0.14
	Total	0.82	0.19
All emotional comments	Individual	0.23	0.21
	Collaborative	0.11	0.14
	Total	0.19	0.19

*Individual group *n* = 49; collaborative group *n* = 30; *NTotal* = 79.*

*Relative frequencies in ratio to all on-task units.*

**TABLE 6:** Multivariate ANOVA to test for differences between the experimental conditions, regarding the relative frequencies of cognitive and emotional reflection around the engagement with conflicting information and the pandemic in general, as well as between all on-task and all off-task comments.

Source	Relative frequencies of	Type III sum of squares	*df*	*Mean Square*	*F*	*p*	η^2^*part*
Experimental conditions	Cognitive comments on text	0.00	1	0.00	0.00	0.97	<0.01
	Cognitive comments beyond text	0.18	1	0.18	3.81	0.06	0.05
	Cognitive comments on pandemic without evidence	0.86	1	0.86	18.61	<0.00	0.20
	Emotional comments on text	0.16	1	0.16	6.84	0.01	0.08
	Emotional comments beyond text	0.01	1	0.01	1.56	0.22	0.02
	All off-task comments	2.15	1	2.15	379.07	<0.00	0.83
	All on-task comments	2.15	1	2.15	379.07	<0.00	0.83
	All cognitive comments	0.26	1	0.26	7.74	0.01	0.09
	All emotional comments	0.26	1	0.26	7.74	0.01	0.09

*Relative frequencies in ratio to all on-task units.*

Furthermore, the group_*coll*_ significantly more often made comments related to the management and coordination of the task (i.e., relative frequencies of off-task units in relation to overall units) (*M* = 34.15%, *SD* = 12.2), compared to those in the group_*in*_ (*M* = 0%, *SD* = 0.0), *F*(1,76) = 379.07, *p* < 0.001, η^2^ = 0.83. Descriptive statistics related to the relative frequencies of types of reflections are presented in Table 5. The results of the multivariate ANOVA to test for differences between the experimental conditions regarding the relative frequencies of cognitive and emotional reflection are presented in Table 6.

### Results in Terms of Information Overload, Information Sourcing Self-Efficacy, and Coping Strategies

We conducted a multivariate generalized linear model, including the between factor *experimental condition* and the within factor *time of measurement* with the dependent variables (1) information overload, (2) information sourcing self-efficacy, and (3) active as well as avoidance coping strategies.

In terms of participants’ reported information overload, the analysis revealed a significant main effect of *time* and no significant main effect of *experimental conditions*, as well as no significant interaction effect between time and experimental conditions. Overall, participants reported higher information overload after the reflection task (*M*_*pre*_ = 2.52, *SD*_*pre*_ = 0.62; *M*_*post*_ = 2.64, *SD*_*post*_ = 0.60; *F*(1, 105) = 8.52, *p* = 0.004, η^2^ = 0.08) (see Table 4).

In regard to participants’ reported information sourcing self-efficacy, the analysis again revealed a significant main effect of *time* and no significant main effect of *experimental conditions*, as well as no significant interaction effect between time and experimental conditions. Overall, participants reported lower self-efficacy after the reflection task (*M*_*pre*_ = 3.48, *SD*_*pre*_ = 0.52; *M*_*post*_ = 3.36, *SD*_*post*_ = 0.57; *F*(1, 105) = 11.19, *p* = 0.001, η^2^ = 0.10) (see Table 4).

Similarly, with reference to the active coping strategies reported by the participants, the analysis revealed a significant main effect of *time* and no significant main effect of *experimental conditions*, as well as no significant interaction effect between time and experimental conditions. All participants reported higher active coping strategies after the reflection task (*M*_*pre*_ = 3.29, *SD*_*pre*_ = 0.78; *M*_*post*_ = 3.45, *SD*_*post*_ = 0.79; *F*(1, 105) = 6.34, *p* = 0.01, η^2^ = 0.06). In terms of participants’ self-reported coping as avoidance, there were no significant differences between time and experimental groups, as well as in terms of an interaction of time and experimental conditions (see Table 4).

## Discussion

### Main Findings

The findings of the present study shed light on how students reflect about their engagement with conflicting scientific information regarding COVID-19 during the beginning of the pandemic, when they had to handle many (conflicting) pieces of information about COVID-19 and had to cope with feelings of uncertainty, loneliness, stress, and anxiety caused by the pandemic (e.g., lockdown). Under these circumstances, the study investigated whether students’ individual and collaborative reflection about their engagement with conflicting information on COVID-19 testing had different impacts on their perceived information overload, their self-efficacy in sourcing information about COVID-19, their coping strategies, as well as their reflective communication behavior. Thus, the study used a combination of quantitative and qualitative data to assess not only important aspects of students’ cognitive processing of information but also their emotions caused by conflicting information.

First, the study provides insights into participants’ cognitive engagement with the information (RQ1).

As we analyzed the content of participants’ reflection around their engagement with the conflicting information in the texts, this study provides insights about the frequencies with which participants engaged in reflections about the evidence in and beyond the texts and the frequencies with which they discussed the pandemic in general (see Supplementary Material 4 for coding scheme). Interestingly, taking all the comments together, participants more often discussed at the cognitive level when they reflected on their engagement collaboratively. The reason for this might be that the participants in the collaborative group more often discussed aspects of the pandemic in general but without referring to specific evidence to support either an opinion or statement (e.g., they more often discussed the government’s pandemic-related measures). In this vein, for instance, speaker B (see excerpt of the communicative reflection behavior presented in Table 2) responded to the comment of speaker A who shared her/his thoughts about any economic or health consequences due to the measures of COVID-19, and thus, speaker A and B both discussed aspects related to the pandemic in more general. In line with approaches on collaborative learning and argumentation, a collaborative setting may support the exchange of multiple views on a topic, having led this study’s students to talk about information related to the pandemic in general (Noroozi et al., 2012). While this may have helped the students in the collaborative condition exchange at an informational level–which might be a form of support seeking (Barak et al., 2008)–they did not more often cognitively engage in discussions about the evidence in the texts, nor beyond the texts. This is contrary to previous findings (e.g., Kuhn and Moore, 2015; Mayweg-Paus and Macagno, 2016). Notably, the critical reasoning of evidence is an important aspect of competently reflecting on the quality of information (Chin and Osborne, 2008; Kuhn and Moore, 2015; Mayweg-Paus and Macagno, 2016; Hendriks et al., 2020) as well as of providing reasons for one’s own and another’s argument (Mayweg-Paus and Macagno, 2016).

Additionally, we assessed two further indicators for participants’ cognitive engagement with conflicting information about COVID-19: With respect to participants’ perceived information overload as well as their self-efficacy in sourcing information about COVID-19, interestingly, participants in both the individual and collaborative groups reported higher information overload and lower self-efficacy after the reflection task, but without any significant differences between the experimental conditions. As participants in the two experimental conditions did not differ in terms of their reported self-efficacy, the reflection task may have led both groups of participants to feel less competent around sourcing information about COVID-19 in general. This is contrary to previous findings on students’ information sourcing self-efficacy, where participants perceived their self-efficacy as higher after reflecting on students’ handling of online information, regardless of whether they were reflecting in an individual or collaborative reflection setting (Zimmermann and Mayweg-Paus, 2021). According to our earlier argumentation (Zimmermann and Mayweg-Paus, 2021), future research is needed to investigate whether reflecting on one’s own competencies (such as inducted by a reflection task) ultimately leads one to perceive their own competencies more realistically or in a more biased manner, leading to an under- or overestimation of oneself. Furthermore, studies indicate that the more information people read, the higher their perceived information load might be (Hong and Kim, 2020; Motta Zanin et al., 2020; Mohammed et al., 2021). In this sense, merely reading additional (new) information presented during the study may have increased participants’ perceived information overload.

Second, the study provides insights into participants’ emotional engagement with the information (R2). Taking together all the emotional comments from the written reflections, those participants who reflected individually more often made emotional comments compared to those participants who reflected collaboratively. The reason for this is that participants in the individual group more often reflected on how the evidence read in the text affected them emotionally (e.g., they more often stated that the information in the texts led to uncertainty or confusion). In this sense, for instance, person A (see example of individual reflection in Table 1), as well as other participants in the individual reflection tasks, explained his/her feelings when reading such contradicting information about COVID-19 and expressed that he/she felt overwhelmed and had mixed feelings about the trustworthiness of the provider of such information.

In terms of participants’ active coping strategies as another indicator of participants’ emotional engagement, all participants reported after the reasoning task that they perform more active coping strategies (i.e., that they ask friends for help or express their feelings to someone they respect and trust when reading unpleasant information about COVID-19), no matter whether they reflected collaboratively or individually about their engagement with the information. This is interesting, as participants in the collaborative as well as individual setting may have become able to activate a set of potential active coping strategies after they reflected on how they engage with conflicting information. First, in the individual reflection group, participants more frequently discussed their stress and confusion caused by the texts, which might have made them more clearly realize that they need to cope with these emotions. Further, in the collaborative reflection group, participants more frequently discussed the pandemic in general, which again might have made them realize that they need to cope with the pandemic in general. Overall, however, future research is needed to investigate what exactly activates students’ reported use of coping strategies.

In this study, we assessed students’ self-reported self-efficacy in sourcing online information about COVID-19, their perceived information overload, their reported coping strategies, as well as their emotional and cognitive engagement with information by analyzing their communicative reflection behavior during the highly externally valid circumstances of the beginning of the pandemic. Yet, even though the time the study was conducted represents a realistic situation for investigating students’ engagement with conflicting online information about science, neither experimental communicative setting was shown to impact the relevant measures for assessing indicators related to students’ information competences (i.e., self-efficacy in sourcing information, information overload) or related to students’ emotional regulation (i.e., coping in a stressful situation because of reading conflicting information). In fact, in terms of students’ rather medium reported negative affect, the findings of the present study contradicted our expectation about students’ negative affective state during such a challenging time of a pandemic (c.f., Wang et al., 2020).

In sum, in terms of the opportunities and challenges of collaboration for students’ cognitive as well as emotional engagement with conflicting information during a reflection task, this study’s findings indicate that only students’ written reflections were influenced by the way participants reflected (individually or collaboratively), whereas for participants in both groups, being presented with new information and performing the reflection task itself increased their perceived information overload, decreased their self-efficacy in sourcing information on COVID-19, and increased their activation of active coping strategies.

### Limitations

With respect to the measurements of the study, the scale to assess participants’ perceived information overload was of low internal consistency, indicating that the items were inconsistent with one another and probably measuring different aspects of or related to information overload (e.g., the perceived overload of information or the perceived overload of recommendations read in the information). Thus, the results in terms of an increased perceived information overload after the reflection task should be interpreted by having the limitation of this measurement in mind: This means that future research may focus on the investigation of whether the validity of measures related to perceived information overload is effected by different types of information (e.g., information about COVID-19 or diet information) or the complexity of the situation in which someone read such information (e.g., during a crisis). In addition, future research may consider additional aspects that have shown to be relevant for students’ emotional engagement during such challenging times (e.g., students’ concerns about any risk of infection: Wang et al., 2020) as well as a diverse methodological approach to assess persons’ emotional engagement–beyond their self-reported assessments or analysis of written reflections (e.g., facial physiology such as EMG measures). In this sense, future research is needed to conclusively explain the relation between persons’ emotional engagement processes when reading (conflicting or unpleasant) scientific information that can be used to derive important actions for their personal life. However, it is interesting to see that when bringing in the qualitative data (in addition to the quantitative data), participants in the individual setting in particular often reflected their uncertainty due to reading the conflicting texts. This may indicate that it is important to assess emotional engagement with information while focusing on specific aspects (e.g., uncertainty when reading information, as well as anxiety due to social measures supported by the information).

While the topic of testing for the virus was highly relevant for students during the pandemic–because students as well as all citizens needed to establish scientifically grounded opinions about COVID-19 information in general–our study only focused on one of many topics. This means that our findings cannot necessarily be generalized. Therefore, future research could expand the picture of students’ emotional as well as cognitive engagement with online scientific information by focusing on different topics.

### Implications

Despite these limitations, this study contributes to research on multiple text comprehension and has further implications for future research and for university students’ education.

Taken together, the findings of this study clearly emphasize the importance of also considering–beyond cognitive processes–students’ emotional engagement with science-related online information. In line with previous research that emphasizes the importance of considering emotional aspects when processing information (e.g., List and Alexander, 2017; Mason et al., 2017), in this study students showed cognitive as well as emotional engagement with conflicting information. In future research, it appears valuable to examine the differences the two forms of processing may have, their interfaces, and whether they are relevant in different ways for different situations. Interestingly, the collaborative and individual reflection settings led students to differently often referred to emotional or cognitive aspects in connection with their engagement with the information. Thus, future research aiming to consider emotional processes of engaging with online information may also investigate how different forms of communicative settings (i.e., collaborative and individual reflection tasks) can help to take both aspects into account (e.g., through interventional studies that examine how instructions can help to increase students’ critical reflection about information on the cognitive as well as emotional level and in diverse communicative settings). Importantly, in this study neither the individual nor the collaborative group received any additional instructions on how to reflect effectively. Thus, future research may investigate whether any instructional support would guide collaborative communication processes more effectively (e.g., with regular instructions on how to focus on the task, on how to consider that everyone is ready to move on, or on how to question the other’s arguments constructively and critically: Noroozi et al., 2012; Mayweg-Paus et al., 2016).

Considering the complexity of processes for engaging with online scientific information–as mentioned above (e.g., List and Alexander, 2017), as well as when engaging collaboratively in discussions with others (e.g., Kuhn and Moore, 2015; Mayweg-Paus and Macagno, 2016)–this study focused on those processes related to participants’ cognitive as well as emotional reflections after they were confronted with only two articles that provided conflicting information. While research focusing on the investigation of students’ actual sourcing of scientific information on the Internet (e.g., Zimmermann and Mayweg-Paus, 2021) may increase the external validity, as it may represent search processes wherein more diverse information can be found in a more valid way, in this study, we used a fictitious scenario in which we presented only two prepared online articles. In this sense, we controlled for the influence of any differences in the found information thus increased the internal validity in terms of students’ evaluation of the read information. However, the study was conducted during a highly confusing social situation–the beginning of the pandemic. Hence, this possibly means that we were nevertheless unable to take other possible influencing factors into account. Future research may additionally investigate students’ engagement with such information under more controllable situations by also considering other aspects: For instance, the results of a recent study on persons’ mental health during COVID-19 lockdown showed that personality traits (e.g., extraversion and neuroticism) were strongly related to psychological well-being during the pandemic (Osimo et al., 2021). Thus, future research may consider personality traits too when investigating whether any personality differences among students may also determine students’ engagement with conflicting information.

In terms of the practical implications for students’ education, the findings reveal that dealing with conflicting science-related information in the context of a crisis seems to be particularly challenging for students (e.g., as all participants reported higher information overload after the reflection task). Consequently, they might need additional guidance and support while engaging with such information, either individually or collaboratively. Our findings provide first insights into what aspects of students’ reflection behavior hold potential for being addressed explicitly in training on digital competence or as an additional support function in social media contexts. First, to help students develop reflection skills for both levels of information processing–the emotional as well as cognitive level–, specific attention must be paid to the communicative settings when discussing digital competence in higher education (Carretero et al., 2017). In this study, for instance, participants in the individual setting more often reflected their uncertainty due to reading the conflicting texts, while participants in the collaborative condition more often reflected the pandemic in general. Thus, university educators as well as students themselves may consider both forms of communicative settings to reflect on the multiple aspects that could ultimately lead to a more competent sourcing of information.

Furthermore, the effect of collaborative reflection on participants’ active coping strategies points to the supporting function of online communities in times of a crisis (for school contexts, Borup et al., 2020) as well as to the pivotal role of social presence in online contexts more generally (see also Richardson et al., 2017). Thus, it would be possible to specifically implement such collaborative support structures, which on the one hand take into account the (1) emotional states of the students and (2) how to provide adequate interventions (e.g., inform about that reading conflicting information may lead to confusion and stress and show possibilities on what to do to reduce this confusion). Accordingly, this would raise the question of how students’ emotional states as well as their cognitive capabilities can be assessed adequately and how they can be approached individually in such interventions.

As we might all agree, studying at university goes beyond academic learning and skill development in a specific domain; rather, universities typically serve the additional function of creating a space for social interaction and exchange regarding topics that are important for students’ everyday life contexts. This understanding suggests that it is critical to implement social spaces in online learning environments at universities that go beyond collaborative learning in official courses of the curriculum. However, students do not seem to automatically profit from the mere presence of another person with regard to emotional coping; thus, educators should be highly attentive to how to match students in social groups and should also be aware of their own role in shaping social support settings or interventions on how to promote students’ successful cognitive as well as emotional engagement with online information.

## Data Availability Statement

The raw data supporting the conclusions of this article will be made available by the authors, without undue reservation.

## Ethics Statement

Ethical review and approval was not required for the study on human participants in accordance with the local legislation and institutional requirements. The patients/participants provided their written informed consent to participate in this study.

## Author Contributions

EM-P and MZ conceived and presented the idea of the study, analyzed the data, were supported by CL in the analysis of data, and equally took the lead in writing the manuscript. CL was responsible for data collection. All authors engaged in writing sections of the manuscript and provided feedback and ideas.

## Conflict of Interest

The authors declare that the research was conducted in the absence of any commercial or financial relationships that could be construed as a potential conflict of interest.

## Publisher’s Note

All claims expressed in this article are solely those of the authors and do not necessarily represent those of their affiliated organizations, or those of the publisher, the editors and the reviewers. Any product that may be evaluated in this article, or claim that may be made by its manufacturer, is not guaranteed or endorsed by the publisher.
