# Supplementary material for: “You Are Not Alone”–Opportunities and Challenges for University Students’ Collaborative Engagement When Dealing With Online Information About COVID-19
Source: Front Psychol. 2021 Oct 5;12:728408. doi: 10.3389/fpsyg.2021.728408 (PMC8524057; doi:10.3389/fpsyg.2021.728408)
Supplement: Supplementary file 1 [file Data_Sheet_1.docx]

Electronic Supplementary Material 1. Instructions

# Original Language and translated version

# Instruction collaborative reflection task (German)

Stellen Sie sich folgende Situation vor:

Das neuartige Corona-Virus "SARS-CoV-2" hält die Welt seit Beginn des Jahres 2020 in Atem. Täglich gibt es neue Meldungen zur Ausbreitung des Erregers und zu Maßnahmen die weltweit zur Eindämmung der Verbreitung des Virus eingesetzt werden. Vielerorts bestehen bereits umfassende Ausgangssperren, Kontaktverbote und Geschäftsschließungen, welche unsere Gesellschaft vor große Herausforderungen stellen und deren Folgen noch nicht absehbar sind.

Sie haben einen aktuellen Beitrag zu den Tests zu COVID-19 gelesen – der Krankheit, die durch das Virus ausgelöst wird. Der Bericht macht Sie neugierig und Sie interessieren sich nun mehr für diese Tests. Daher recherchieren Sie noch weiter. Zwei Online-Artikel zum Thema "Corona-Tests" haben dabei ihr besonderes Interesse geweckt.

Ein*e Freund*in scheint ebenfalls auf diese beiden Texte aufmerksam geworden zu sein und sendet Ihnen die selben Texte über ihren gemeinsamen Chat zu. Gemeinsam beschließen sie sich über die beiden Texte auszutauschen. Auf der nächsten Seite finden Sie diese beiden Online-Artikel. Klicken Sie auf "Weiter

---

Lesen Sie sich bitte die folgenden Informationen durch. Es handelt sich dabei um zwei echte Online-Artikel, die jeweils Ende März 2020 im Zusammenhang mit der Corona-Krise veröffentlicht wurden. Sie sehen hier die pdf-Ansicht beider Online-Artikel. Bitte lesen Sie sich die Texte aufmerksam durch und klicken erst auf "Weiter", wenn Sie damit fertig sind!

---

Bitte besprechen Sie gemeinsam im Chat, wie Sie mit solchen widersprüchlichen Informationen umgehen. Wie geht es Ihnen, wenn Sie solche widersprüchlichen Informationen lesen? Wie versuchen Sie damit umgehen? Ihr gemeinsames Ziel im Gespräch ist es, sich gegenseitig beim reflektieren zu unterstützen! Dabei gibt es kein "Richtig" oder "Falsch". Aus anderen Untersuchungen wissen wir, dass Menschen dafür etwa 30 Minuten Zeit benötigen. Sobald sie das Gefühl haben, dass Sie beide ausreichend Zeit hatten, um über Ihren Umgang mit solchen Informationen zu sprechen, klicken Sie aus "Weiter". Starten Sie jetzt ihr Gespräch im Chat!

# Instruction collaborative reflection task (Translation)

Imagine the following situation:

The world has been holding its breath since the new corona virus "SARS-CoV-2" appeared at the beginning of 2020. Every day there are new reports about the spread of the pathogen and containment measures that are used worldwide. In many places there are already extensive exit locks, contact bans and shop closings, which pose great challenges for our society, but the consequences of these actions are not yet foreseeable.

You have read a recent post on testing for COVID-19 – the disease caused by the virus. The report aroused your curiosity and made you more interested in these tests. Therefore, you do further research. Two online articles on the subject of "Corona tests" caught your particular interest.

A friend also seems to have noticed these two texts and is sending you the same texts via your common chat. Together you decide to talk about the two texts. On the next page you will find these two online articles. Click on Continue

---

Please read the following information. These are two real online articles that were published at the end of March 2020 in connection with the Corona crisis. You can see the PDF view of both online articles here. Please read the texts carefully and only click on "Next" when you are finished!

---

Please discuss together in the chat how to deal with such conflicting information. How do you feel when you read such conflicting information? How do you try to deal with it? Your common goal in the conversation is to support each other in reflecting! There is no such thing as "right" or "wrong". We know from other studies that people need about 30 minutes for this. Once you feel that the two of you have had sufficient time to discuss how you are handling such information, click Next. Start your conversation in the chat now!

**Instruction individual reflection task (German)**

Das neuartige Corona-Virus "SARS-CoV-2" hält die Welt seit Beginn des Jahres 2020 in Atem. Täglich gibt es neue Meldungen zur Ausbreitung des Erregers und zu Maßnahmen die weltweit zur Eindämmung der Verbreitung des Virus eingesetzt werden. Vielerorts bestehen bereits umfassende Ausgangssperren, Kontaktverbote und Geschäftsschließungen, welche unsere Gesellschaft vor große Herausforderungen stellen und deren Folgen noch nicht absehbar sind.

Sie haben einen aktuellen Beitrag zu den Tests zu COVID-19 gelesen – der Krankheit, die durch das Virus ausgelöst wird. Der Bericht macht Sie neugierig und Sie interessieren sich nun mehr für diese Tests. Daher recherchieren Sie noch weiter. Zwei Online-Artikel zum Thema "Corona-Tests" haben dabei ihr besonderes Interesse geweckt.

Auf der nächsten Seite finden Sie diese beiden Online-Artikel. Klicken Sie auf "Weiter"!

---

Lesen Sie sich bitte die folgenden Informationen durch. Es handelt sich dabei um zwei echte Online-Artikel, die jeweils Ende März 2020 im Zusammenhang mit der Corona-Krise veröffentlicht wurden. Sie sehen hier die pdf-Ansicht beider Online-Artikel. Bitte lesen Sie sich die Texte aufmerksam durch und klicken erst auf "Weiter", wenn Sie damit fertig sind!

---

Bitte erörtern Sie, wie Sie mit solchen widersprüchlichen Informationen umgehen. Wie geht es Ihnen, wenn Sie solche widersprüchlichen Informationen lesen? Wie versuchen Sie damit umgehen?

Ihr Ziel ist es dabei, sich selbst zu reflektieren! Dabei gibt es kein "Richtig" oder "Falsch". Aus anderen Untersuchungen wissen wir, dass Menschen dafür etwa 30 Minuten Zeit benötigen. Sobald sie das Gefühl haben, dass Sie ausreichend Zeit hatten, um Ihren Umgang mit solchen Informationen zu beschreiben, klicken Sie aus "Weiter". Geben Sie hier ein, wie Sie mit solchen Informationen umgehen und wie Sie sich dabei fühlen.

**Instruction individual reflection task (Translation)**

The world has been holding its breath since the new corona virus "SARS-CoV-2" appeared at the beginning of 2020. Every day there are new reports about the spread of the pathogen and containment measures that are used worldwide. In many places there are already extensive exit locks, contact bans and shop closings, which pose great challenges for our society, but the consequences of these actions are not yet foreseeable.

You have read a recent post on testing for COVID-19 – the disease caused by the virus. The report aroused your curiosity and made you more interested in these tests. Therefore, you do further research. Two online articles on the subject of "Corona tests" caught your particular interest.

On the next page you will find these two online articles. Click on Continue

---

Please read the following information. These are two real online articles that were published at the end of March 2020 in connection with the Corona crisis. You can see the PDF view of both online articles here. Please read the texts carefully and only click on "Next" when you are finished!

---

Please discuss how to handle such conflicting information. How do you feel when you read such conflicting information? How do you try to deal with it?

Your goal is to reflect on yourself! There is no such thing as “right” or “wrong.” We know from other studies that people need about 30 minutes for this. As soon as you feel that you have had enough time to describe how you deal with such information, click on "Next". Enter here how you will deal with such information and how you feel about it.
