# Supplementary material for: “You Are Not Alone”–Opportunities and Challenges for University Students’ Collaborative Engagement When Dealing With Online Information About COVID-19
Source: Front Psychol. 2021 Oct 5;12:728408. doi: 10.3389/fpsyg.2021.728408 (PMC8524057; doi:10.3389/fpsyg.2021.728408)
Supplement: Supplementary file 2 [file Data_Sheet_2.docx]

Electronic Supplementary Material 2. Text A in original language and translated version.

**Praxis für Allgemeinmedizin**

Dr. med. Manfred Kuhnfried

Dr. med. Manfred Kuhnfried leitet seit 1983 seine Praxis für Allgemeinmedizin. Er hat folgende Ausbildungen absolviert: Studium Humanmedizin, Universität Münster 1974 -1980 / Klinische Ausbildung Innere Medizin

Das Corona Virus hat unser Leben fest im Griff. Schulen und Kitas schließen. Demnächst gibt es bestimmt auch eine komplette Ausgangssperre. Dies ist eine stressige Zeit. Nicht nur für meine Praxis, sondern auch für alle anderen Praxen und Krankenhäuser in Deutschland. Vor allem ist es eine stressige Zeit, weil Patienten anrufen und sich testen lassen oder weil sie sich gegen Pneumokokken impfen lassen wollen.

**Was wir über die Tests wissen**

Viele Menschen sind verunsichert und wollen sich deswegen testen lassen. Ob diese Tests allerdings tatsächlich sinnvoll sind ist nicht wissenschaftlich abgesichert.

Wir Ärzte dürfen entscheiden, welche Patienten getestet werden. Das an sich bedeutet schon mal, dass wir aufgrund der Kapazität natürlich nur die Personen testen, die auch Symptome aufweisen oder im Kontakt zu einer infizierten Person standen. Das bedeutet also, wenn sie auch potentiell Kontakt zu den Viren hatten. Wenn wir testen, wird den Patienten ein Rachen–Nasenabstrich entnommen. Teilweise wird auch ausgehustetes Sekret aus den Bronchien verwendet. Darin werden bestimmte Gen-Bruchstücke des Coronavirus (RNA) gesucht. Da die Proben mengenmäßig für eine Bestimmung nicht ausreichen, werden diese zunächst mehrmals vervielfältigt. Dies geschieht durch die Polymerase-Kettenreaktions-Methode (englisch "Polymerase Chain Reaction" oder kurz PCR). Die Analyse des Probenmaterials soll dann zeigen, ob die gesuchte Gensequenz des Virus in der Probe vorhanden ist oder nicht. Das bringt aber eine gewisse Unschärfe mit sich.

**Fachleute halten die Wirksamkeit der Tests für fraglich**

Wang Chen ist Präsident der „Chinese Academy of Medical Sciences“. Er beziffert die Genauigkeit des Coronavirus Tests auf 30 bis 50%. Der Test erkennt also nur bei jedem zweiten oder dritten Infizierten, dass dieser wirklich infiziert ist. Allerdings gibt es nicht nur bei der Ermittlung der Infizierten eine große Fehlerquote. Auch sehr viele Gesunde werden fälschlicherweise so eingestuft als wären sie mit dem Coronavirus infiziert. Diese Menschen werden dann aufgrund dieser sogenannten falsch positiven Tests falsch behandelt und sorgen zudem für eine viel zu hohe Anzahl an infizierter Menschen in den Statistiken. Eine aktuelle wissenschaftliche Studie vom 5. März 2020 schätzt, dass vier von fünf getesteten Menschen (also in etwa 80%) fälschlicherweise als coronapositiv eingruppiert werden. Laut BBC-Berichten wurden teilweise Patienten bis zu sechs Mal mit dem Resultat „Corona negativ“ getestet bis dann im siebten Versuch doch ein positives Ergebnis herauskam. Augenscheinlich wurde hier so lange getestet bis schließlich ein Corona positives Ergebnis vorlag.

Die Infektiologin Prof. Isabella Eckerle ist Leiterin des Zentrums für Viruserkrankungen an der Universität Genf. Sie weist auf Aussagekraft über infektiöse Viren hin. Demnach lässt sich bei den verwendeten COVID-19 Tests nur Virus-Genmaterial nachweisen. Das wiederum sagt aber nichts über das Vorhandensein oder die Anzahl an infektiöser und somit „aktiver“ Viren aus. Selbst Kary B. Mullis (der Entwickler des PCR-Tests und Träger des Nobelpreises in Chemie) hält den PCR-Test für untauglich, um Viren zu identifizieren.

**Was bedeutet das für die Maßnahmen der Regierung**

Ich habe bereits in meinem letzten Artikel über die gesamte mediale Berichterstattung rund um das Coronavirus berichtet. Ich kann verstehen, dass viele Menschen besorgt sind. Ich kann auch verstehen, dass schnell Maßnahmen ergriffen werden mussten. Allerdings bezweifle ich, dass dies wirklich sinnvolle Maßnahmen sind. Dabei ist mein wichtigstes Argument, dass diese gesellschaftlichen Maßnahmen möglicherweise aufgrund von Ergebnissen eines fragwürdigen Tests getroffen werden. Ich finde es erschreckend, dass diesem Test anscheinend blind vertraut wird.

Ich würde sogar noch weiter gehen und auch in Frage stellen, ob auch weitere Maßnahmen, wie zum Beispiel das öfter genannte Contact Tracing wirklich sinnvoll sind. Bei dieser Maßnahme werden Personen isoliert, wenn sie positiv auf das Virus getestet wurden. Zusätzlich müssen dann in diesem Fall auch die Kontaktpersonen isoliert werden. Bei einer so hohen Zahl an falschen Testergebnissen würden tausende Menschen zu Unrecht in Quarantäne kommen.

Vermutlich kann zum jetzigen Zeitpunkt niemand genau absehen, welche dauerhaften Auswirkungen diese vielleicht überflüssigen Maßnahmen auf unsere Gesellschaft haben werden. Vielleicht schaden sie uns sogar mehr, als dass sie uns eigentlich nützen sollen. Wir wissen nicht, ob wir durch fehlendes Hinterfragen der Evidenz der Test-Diagnostik wohlmöglich Hunderttausende Freiberufler und kleine Unternehmer in die dauerhafte Armut geschickt haben. Staatliche Hilfsprogramme werden dies vielleicht niemals auffangen können. Vor allem wenn man die Masse an Anfragen dazu bedenkt. Was mir am Ende bleibt ist Unverständnis darüber, wie wir die Aufgabe unserer Freiheit auf Grundlage dieser unsicheren Test-Ergebnisse einfach so hinnehmen konnten. Ich hoffe, wir fangen bald an genau darüber nachzudenken.

# Quellen:

Zhuang et al. Potential false-positive rate among the 'asymptomatic infected individuals' in close contacts of COVID-19 patients, [Chinese Medical Association Publishing House Ltd.](http://journal.yiigle.com/LinkIn.do?linkin_type=pubmed&issn=0254-6450&year=2020&vol=41&issue=4&fpage=485), 05.03.2020 (<https://www.ncbi.nlm.nih.gov/pubmed/32133832>, letzmalig abgerufen am 30.03.2020)

**Practice for general medicine**

Dr. med. Manfred Kuhnfried

Dr. med. Manfred Kuhnfried has been running his general practice since 1983. He has completed the following training: Studies in human medicine, University of Münster 1974 -1980 / clinical training in internal medicine

The Coronavirus is keeping a tight hold on our lives. Schools and daycare centers are closing. Certainly there will be a complete curfew in the near future. This is a stressful period. Not only for my practice, but also for all other practices and hospitals in Germany. Most of all, it's a stressful period because patients are calling and wanting to get tested or vaccinated against pneumococci.

**What we know about the tests**

Many people are insecure and therefore want to be tested. Whether these tests are actually useful, however, is not scientifically proven.

We doctors can decide which patients are tested. That in itself means that due to capacity issues, we only test those people who are showing symptoms or who have been in contact with an infected person. In short, if they had potential contact with the virus.

When we test, a throat-nasal swab is taken from the patient. Sometimes coughed up secretion from the bronchi is also used. Certain gene fragments of the Coronavirus (RNA) are searched for in it. Since the samples are not quantitatively sufficient for a determination, they are first duplicated several times.

This is done by the polymerase chain reaction method (English "polymerase chain reaction" or short PCR). The analysis of the sample material should then show whether the virus gene sequence is present in the sample. But that involves a certain imprecision.

**Experts consider the effectiveness of the tests open to questions**

Wang Chen is President of the Chinese Academy of Medical Sciences. He puts the accuracy of the Coronavirus test at 30 to 50%. The test only detects that every second or third person infected is really infected. However, there is a high error rate not only when identifying the infected. Also, many healthy people are incorrectly classified as if they were infected with the Coronavirus.

According to BBC reports, some patients were tested up to six times with the result "Corona negative" until a positive result came out in the seventh attempt. Apparently, they were testing for too long until a Corona positive result was finally available.

Infectiologist Prof. Isabella Eckerle is head of the Center for Viral Diseases at the University of Geneva. She indicates the informative value of infectious viruses. Accordingly, only virus genetic material can be detected in the COVID-19 tests used. This in turn says nothing about the presence or number of infectious and therefore "active" viruses. Even Kary B. Mullis (the developer of the PCR test and winner of the Nobel Prize in Chemistry) considers the PCR test to be unsuitable for identifying viruses.

**What does this mean for government measures**

In my last article I already reported on the entire media reporting on the Coronavirus. I can understand that many people are concerned. I can also understand that measures had to be taken quickly. However, I doubt that these are useful measures. My main argument is that these social measures may be based on the results of a questionable test. I find it appalling that this test is apparently blindly trusted.

I would go even further and question whether other measures, such as the often-mentioned contact tracing, are useful. This measure isolates people if they test positive for the virus. In this case, the contact persons must be isolated, too. With such a high number of false test results, thousands of people would be unjustly quarantined.

At this point, no one can presumably foresee what lasting effects these perhaps superfluous measures will have on our society. Maybe they will do us more harm than good. We do not know whether we may have sent hundreds of thousands of freelancers and small entrepreneurs into permanent poverty by not questioning the evidence of test diagnostics.

Government aid programs may never be able to compensate for this. Especially when you consider the mass of inquiries. What remains for me, in the end, is a lack of understanding of how we could simply accept giving up our freedom on the basis of these uncertain test results. I hope we will start thinking carefully about this soon.
