# Supplementary material for: “You Are Not Alone”–Opportunities and Challenges for University Students’ Collaborative Engagement When Dealing With Online Information About COVID-19
Source: Front Psychol. 2021 Oct 5;12:728408. doi: 10.3389/fpsyg.2021.728408 (PMC8524057; doi:10.3389/fpsyg.2021.728408)
Supplement: Supplementary file 3 [file Data_Sheet_3.docx]

Electronic Supplementary Material 3. Text B in original language and translated version.

**Klinik für Infektiologie**

Dr. med. Peter Vernaut

Dr. med. Peter Vernaut ist Chefarzt der Infektiologie und seit 1985 im Universitätsklinikum Münster tätig. Vor seiner Arbeit im Klinikum hat er folgende Ausbildungen absolviert: Studium Humanmedizin, Universität Heidelberg 1976 -1982 / Klinische Ausbildung Innere Medizin

**Vielleicht 90% von Coronainfektionen unbemerkt!**

Zurzeit sind wir maximal beschäftigt mit der Bewältigung unserer Aktivitäten zur Vorbereitung der noch kommenden Ausbreitung von COVID-19 in der Republik. Die politischen Entscheidungen der letzten Wochen waren teilweise überstürzt. Das war auch notwendig, denn in solchen Situationen dürfen wir keine Zeit verpassen. Doch es lohnt sich auch – von Zeit zu Zeit das – was wir gerade tun zu reflektieren.

**Follow the Science!**

Mit diesem Zitat hat Barack Obama in seiner Präsidialzeit ein klares Zeichen gesetzt: Er hat klar gemacht, dass politische Entscheidungen letztendlich immer auf Evidenz abgestützt sein müssen. Und er hat seine besten Wissenschaftler immer wieder zu Beratungen zugezogen. Eine Haltung, welche im Moment sträflich vernachlässigt wird. Wir sind in einem Zustand, in dem der Einbezug von Fachkompetenz in den politischen Entscheidungen rund um Corona kaum noch Platz hat. Das Tagesgeschäft lässt Reflexion fast nicht mehr zu.

**Bahnbrechende Arbeit von den Medien kaum beachtet**

So wundert es nicht, dass die Publikation von Li et al. fast unbemerkt an uns vorbei ging. Doch die Arbeit hat es in sich.

Die Epidemiologen haben die Ausbreitung von COVID-19 in China untersucht und sind der Frage nachgegangen, wie oft denn die neuen Infektionen überhaupt entdeckt wurden. Die Methodik ist ausgeklügelt, sie basiert auf Berechnungen von Kontaktwahrscheinlichkeiten und bezieht auch die Mobilitätsdaten und weitere komplexe Informationen ein.

Das Resultat ist jedoch recht einfach erzählt:

Rund 85% (82-90%) aller Infektionen sind erfolgt, ohne dass jemand die Infektion bemerkt hat

Etwa 55% der unbemerkt Infizierten haben weitere Personen angesteckt

Eigentlich vermuten wir dies schon lange. Anders wäre es gar nicht erklärbar gewesen, dass die Infektionszahlen in China nach einigen Wochen gegen Null gesunken sind. Denn mit einer Immunitätsrate von weniger als 1% der Bevölkerung (d.h. weniger als 100.000 von rund 10 Mio. Personen in Wuhan sind immun) kann man das nicht erklären. Es würde nicht mal verwundern, wenn weitere Untersuchungen die Rate von stummen Infektionen noch höher beziffern werden.

**Konsequenzen für Epidemiologie, Fallmanagement und Politik**

Diese neue und nicht überraschende Erkenntnis muss rasch zu einer evidenzbasierten Korrektur unserer Maßnahmen führen.

**1. Stoppt die wilde Testaktivität!**
Zunächst einmal ist klar, dass wir aufhören müssen, COVID-19 Tests zu machen. Diese Tests nützen niemandem etwas, weil sie nur den kleinsten Teil der Ansteckenden entdecken. Dazu sind die Kosten mit etwa 200.- Euro pro Test prohibitiv hoch. Das ist ineffizient. Tests helfen uns im Krankenhaus Patienten mit schweren Infektionen zu behandeln. Dort wollen wir wissen, wer infiziert ist. Doch um die Ausbreitung zu stoppen, bringt es nichts, wie die Arbeit von Li et al., 2020 gezeigt hat.

**2. Überlegt die nächsten Schritte!**
Ich habe schon erwähnt: Sofortmaßnahmen müssen sofort sein. Das liegt in ihrer Natur. Doch dann braucht es wieder Phasen der Besonnenheit und des Nachdenkens.

Dafür hilft es sich ein weiteres Ergebnis der oben erwähnten Studie anzusehen: Am allerwichtigsten bei Corona ist es, Erkrankte zu isolieren und ihre Kontaktpersonen nachzuverfolgen (das sogenannte Contact Tracing). Alle weiteren momentan eingeführten Maßnahmen sind weniger erfolgsversprechend. Doch auch das Contact Tracing hat seine Tücken. Im Schnitt müssten bei Corona für jeden Infizierten 36 weitere Personen nachverfolgt werden. Bei 150 neuen Infektionen pro Tag – wie z.B. in Berlin – kommen die Gesundheitsbehörden schnell an ihre Grenzen. Das ist der Grund, warum einige Experten raten, soziale Kontakte auch für Gesunde zu beschränken.

Ich halte daher nichts von einer generellen Beendigung der Maßnahmen, ganz im Gegenteil! Wir wissen nicht, wie viele Menschen noch infiziert sind. Da wir nicht alle Menschen testen können ist es wahrscheinlich, dass viele Menschen unerkannt das Coronavirus in sich tragen. Daher ist es wahrscheinlich, dass viele Menschen mit relativ milden Symptomen das Virus auch weiterverbreiten können. Um diese Verbreitung durch stumme Infektionen zu stoppen, brauchen wir auch Maßnahmen wie Ausgangsbeschränkungen. Ich möchte mich jedoch auch für eine stetige Anpassung der Maßnahmen an die neuesten wissenschaftlichen Erkenntnisse einsetzen, da diese erhebliche Auswirkungen für unsere Gesellschaft und Wirtschaft haben, die wir derzeit kaum vorhersehen können.

Daher sollten wir jetzt anfangen darüber nachzudenken: Welche Maßnahmen brauchen wir und zu welchem Zeitpunkt brauchen wir diese!

**Literaturangaben**

[Li R. et al. Substantial undocumented infection facilitates the rapid dissemination of novel coronavirus (SARS-CoV2). Science. 2020](http://www.ncbi.nlm.nih.gov/pubmed/32179701" \t "_blank" \o "Li R. et al. Substantial undocumented infection facilitates the rapid dissemination of novel coronavirus (SARS-CoV2). Science. 2020)

**Infectious Disease Clinic**

Dr. med. Peter Vernaut

Dr. med. Peter Vernaut is the chief physician in infectious diseases and has been working at the Münster University Hospital since 1985. Before working at the clinic, he completed the following training: Studies in human medicine, University of Heidelberg 1976-1982 / clinical training in internal medicine

**Maybe 90% of Corona infections go unnoticed!**

Now we are busy coping with our actions in preparation for the coming spread of COVID-19 in the republic. The political decisions of the last few weeks were partly hasty. That was also necessary, because in such situations we can't waste any time. But it is also worthwhile – from time to time – to reflect on what we are currently doing.

**Follow the science!**

With this quote, Barack Obama sent a clear signal during his presidency: He made it clear that political decisions must ultimately always be based on evidence. And he has repeatedly consulted his best scientists, an attitude that is criminally neglected now. We are in a state in which the inclusion of professional competence in the political decisions relating to Corona hardly has any place. Day-to-day business almost no longer allows for reflection.

**Groundbreaking work barely noticed by the media**

Therefore, it is not surprising that the publication by Li et al. passed us almost unnoticed. However, the work is a class of its own.

Epidemiologists have examined the spread of COVID-19 in China and investigated how often the new infections were discovered in the first place. The methodology is sophisticated, it is based on calculations of contact probabilities and includes mobility data and other complex information.

The result, however, is told quite simply:

• Around 85% (82-90%) of all infections occurred without anyone noticing the infection

• Around 55% of those unnoticed infections have infected other people

We have suspected this for a long time. Otherwise, it would not have been explainable that the number of infections in China fell to zero after a few weeks. Because with an immunity rate of less than 1% of the population (i.e., less than 100,000 out of around 10 million people in Wuhan are immune) one cannot explain this. It would not be surprising if further research puts the rate of silent infections even higher.

**Consequences for epidemiology, case management and politics**

This new and not surprising finding must quickly lead to an evidence-based correction of our measures.

**1. Stop the unfocused testing activity!**

First, it is clear that we need to stop doing COVID-19 tests. These tests are of no use to anyone because they only detect the smallest portion of those who are contagious. In addition, the costs of around 200 euros per test are prohibitively high. It's inefficient. Tests help us treat patients with severe infections in the hospital. There we want to know who is infected. Nevertheless, there is no point in stopping the spread, as the work by Li et al., 2020 has shown.

**2. Think about the next steps!**

I have already mentioned: Immediate measures must be immediate. It's in their nature. Then you need phases of prudence and reflection again.

To do this, it helps to look at another result of the above-mentioned study: The most important thing about Corona is to isolate the sick people and track their contacts (so-called contact tracing). All other currently introduced measures are less promising. However, contact tracing also has its pitfalls. On average, with Corona, 36 additional people would have to be followed up for each infected person. With 150 new infections per day - such as in Berlin - the health authorities quickly reach their limits. This is the reason why some experts advise restricting social contacts even for healthy people.

I therefore do not believe in a general termination of the measures; on the contrary! We do not know how many people are still infected. Since we cannot test all people, it is likely that many people carry the Coronavirus undetected. Therefore, it is likely that many people with relatively mild symptoms can spread the virus as well. To stop this spread through silent infections, we also need measures such as exit restrictions. However, I would also like to advocate constant adaptation of the measures to the latest scientific findings, as these have considerable effects on our society and economy that we can hardly foresee now.

Now, we should start thinking about it: What measures do we need and when do we need them!

**References**

[Li R. et al. Substantial undocumented infection facilitates the rapid dissemination of novel coronavirus (SARS-CoV2). Science. 2020](http://www.ncbi.nlm.nih.gov/pubmed/32179701)
