# Supplementary material for: “You Are Not Alone”–Opportunities and Challenges for University Students’ Collaborative Engagement When Dealing With Online Information About COVID-19
Source: Front Psychol. 2021 Oct 5;12:728408. doi: 10.3389/fpsyg.2021.728408 (PMC8524057; doi:10.3389/fpsyg.2021.728408)
Supplement: Supplementary file 4 [file Data_Sheet_4.docx]

Electronic Supplementary Material 4. Coding Scheme and examples for coding categories

| *Table 1.* Examples for category cognitive comments on text (translation in brackets). | |
| --- | --- |
| Participant’s reasoning behavior | |
| Individual | Collaborative |
| Auch wenn beide Texte von Ärzten stammen, hatte ich eher den Eindruck, dass es um persönliche Meinungen ging und wissenschaftliche Grundlagen nach Belieben herangezogen wurden, um entsprechende Argumente zu untermauern.  (Even though both texts were written by doctors, I rather had the impression that it was about personal opinions and scientific bases were used at will to support corresponding arguments.) | Der zweite Text hat mich dann erst darauf aufmerksam gemacht, dass der Test wohl recht unzuverlässig ist. Da würde ich jetzt eher nochmal hinterherrecherchieren, ob das auch tatsächlich so stimmt.  (The second text then made me aware that the test is probably quite unreliable. I would now rather do more research to find out whether this is actually true.) |
| Widersprüchlichkeit ist in der Berichterstattung für mich keine Überraschung mehr, gerade zum Coronavirus gibt es durch die unterschiedlichsten Gruppen von Personen, die sich mit dem Thema konfrontiert fühlen allerlei Perspektiven und persönliche Erfahrungen, die wiederum verarbeitet, reflektiert und weiter verbreitet werden.  (Contradictions in reporting are no longer a surprise to me; especially with regard to the Coronavirus, there are all kinds of perspectives and personal experiences through the most diverse groups of people who feel confronted with the topic, which in turn are processed, reflected upon and further disseminated.) | Was ich relativ unbeeindruckend bzw. wenig hilfreich finde an den Artikeln, ist der Appell an mehr Nachdenken bzw. mehr Informationen am Ende der beiden Artikel. Sie kritisieren beide die Grundlage auf denen Entscheidungen getroffen werden als unzureichend, ohne aber eine konstruktive Alternative zu bieten  (What I find relatively unimpressive or unhelpful about the articles is the appeal for more thought or more information at the end of both articles. They both criticize the basis on which decisions are made as inadequate, but without offering a constructive alternative.) |
|  | |

| *Table 2*. Examples for category cognitive comments beyond text (translation in brackets). | |
| --- | --- |
| Participant’s reasoning behavior | |
| Individual | Collaborative |
| Parallel würde ich mithilfe von Google versuchen weitere Informationen zum Thema heranzuziehen und wenn diese keine valide/vertrauenswürdige Antwort liefern, würde ich noch Zeit in die Recherche über Google Scholar investieren und mir zumindest weitere Abstracts verschiedener Paper durchlesen um Fragen, die beim Lesen widersprüchlicher Informationen aufkamen, zu klären bzw. meine eigene Einschätzung zu untermauern oder andernfalls zu überdenken.  (In parallel, I would use Google to try to find further information on the topic and if this does not provide a valid/trustworthy answer, I would invest more time in research via Google Scholar and at least read through further abstracts of various papers to clarify questions that arose when reading contradictory information or to support my own assessment or otherwise to reconsider.) | Also, wir haben ja in der letzten Zeit oft widersprüchliche und unseriöse Informationen zum Thema Covid-19 erhalten und ich gehe mittlerweile so damit um, dass ich mir vertrauenswürdige Quellen und Personen heraussuche und eher irrelevante Quellen ignoriere...  (So, we have often received contradictory and dubious information on the subject of COVID-19 in recent times and I now deal with it in such a way that I look for trustworthy sources and people and ignore rather irrelevant sources...) |
| Ich schaue auf welcher Plattform ich diese Artikel gelesen habe und welche Quellen angegeben wurden. Anhand dieser Informationen und anhand dem was ich bereits weiß, versuche ich mir ein Bild davon zu machen für wie vertrauenswürdig ich diese Artikel halte.  (I look at what platform I read these articles on and what sources were cited. Based on this information and what I already know, I try to get an idea of how trustworthy I think these articles are.) |  |
|  | |

| *Table 3.* Examples for category cognitive comments about the pandemic in general (translation in brackets). | |
| --- | --- |
| Participant’s reasoning behavior | |
| Individual | Collaborative |
| Aber ich habe mich schon seit längerer Zeit damit angefreundet, nicht immer alles begründen und wissen zu können. Vor allem wenn es um Corona geht.  (But for some time now, I get used to not always being able to justify and know everything. Especially when it comes to Corona.) | Aber trotz alledem finde ich braucht man noch Zeit um über weitere Verfahren nachzudenken, weil das Virus noch zu unerforscht ist  (But despite all this, I think we still need time to think about further procedures, because the virus is still too unexplored.) |
| Allgemein finde ich es auch fragwürdig, Vorschriften wie Social-Distancing und der teilweise daraus resultierenden Quarantäne so stark zu hinterfragen, anstatt es einfach für eine Zeit lang umzusetzen und abzuwarten, was die Forschung mit der Zeit konkret belegen kann.  (In general, I also find it dubious to question regulations like social distancing and the partially resulting quarantine so strongly, instead of simply implementing it for a while and waiting to see what research can concretely prove over time.) |  |
|  | |

| *Table 4.* Examples for category emotional comments on text (translation in brackets). | |
| --- | --- |
| Participant’s reasoning behavior | |
| Individual | Collaborative |
| Nach dem Lesen der Artikel war ich zunächst verwirrt und habe das Bedürfnis noch einmal selbst nachzurecherchieren.  (After reading the articles, I was initially confused and feel the urge to do some more research myself.) | Ansonsten fühle ich mich auf jeden Fall total unwohl, wenn ich mit solchen Widersprüchlichkeiten konfrontiert bin.  (Apart from that, I definitely feel totally uncomfortable when confronted with such contradictions.) |
| Zunächst einmal herrscht eine große Unsicherheit. Man weiß selber nicht, welcher Artikel als wahr oder falsch anzusehen ist, da diese sich inhaltlich differenzieren.  (First of all, there is a huge uncertainty. One does not know oneself which article is to be regarded as true or false, since they differ in content.) | Es ist immer beängstigend neue, widersprüchliche Infos zu lesen, weil man niemals sicher sein kann, dass sie stimmen oder eben nicht.  (It is always scary to read new, contradictory information, because you can never be sure that it is true or not.) |
|  | |

| *Table 5*. Examples for category emotional comments beyond text (translation in brackets). | |
| --- | --- |
| Participant’s reasoning behavior | |
| Individual | Collaborative |
| Ich mache mir eher Sorgen um das was all dieser enorme Informationsfluss in der Bevölkerung auslöst, als um den Virus selbst.  (I'm more worried about what all this enormous flow of information will do to the population than about the virus itself.) | Also mir geht es mit der Situation verhältnismäßig gut, ich schaffe es mich da emotional nicht zu sehr einnehmen zu lassen. Ein paar Sorgen mache ich mir natürlich schon, insbesondere um meine Großeltern, aber ich bin nicht in Panik oder so ähnlich.  (So, I'm doing relatively well with the situation, I manage not to get too emotionally involved. Of course, I am a bit worried, especially about my grandparents, but I am not panicking or anything like that.) |
|  | Ich habe ebenfalls keine Angst zu erkranken, denke aber häufiger an Personen, die zu den sogenannten Risikogruppen gehören.  (I'm not afraid of getting sick either, but I think more often about people who belong to the so-called risk groups.) |
|  |  |
|  | |
|  | |
